# Microscopic Falsehoods

**Published:** 1882-09

**Authors:** 


### MICROSCOPIC FALSEHOODS.

"Figures won't lie," we are told, but bank cashiers, etc., seem to make them lie quite commonly nowadays. *Apropos* to this, did it never occur to the therapeutic microscopists that they are making the microscope lie most outrageously when they make it assume to tell us of the therapeutic or pathogenetic action of drugs? They ought to, and must know, that it cannot tell the truth on that subject, then what do they mean in forcing it to speak thereon?

ANTI-MICROSCOPY IN THERAPEUTICS.

